# Metastatic Breast Cancer to the Esophagus Causing Pseudoachalasia

**DOI:** 10.14309/crj.0000000000001666

**Published:** 2025-04-04

**Authors:** Garrett Teskey, Alexander Prevallet, Rohit Khanna, Franklin Chung-Han Tsai, Walter James Coyle

**Affiliations:** 1 Division of Internal Medicine, Scripps Mercy Hospital, San Diego, CA; 2 Division of Gastroenterology & Hepatology, Riverside Community Hospital, Riverside, CA; 3 Division of Gastroenterology & Hepatology, Scripps Clinic, La Jolla, CA

**Keywords:** pseudoachalasia, Endosonograpgy, EUS, esophageal metastasis

## Abstract

A unique case involving a 65-year-old woman with recurrent hormone-positive, HER-2-negative breast cancer stage IV (T2N2M1) with known metastases to the bone presented with worsening dysphagia and esophageal wall thickening on imaging masquerading as achalasia. However, endosonography (EUS)-guided biopsy of the esophagus wall demonstrated metastatic breast cancer to the esophagus causing pseudoachalasia.

## INTRODUCTION

Esophageal achalasia, a rare condition affecting approximately one case per 100,000 individuals annually, is characterized by the absence of esophageal contractility and failure of lower esophageal sphincter relaxation.^1^ However, pseudoachalasia due to secondary benign or malignant causes can present with similar findings. Pseudoachalasia is diagnosed in 2%–4% of individuals presenting with achalasia.^2^ Conditions which can be associated with pseudoachalasia include peptic strictures, postsurgical changes, and tumors.^3^ While the clinical, radiologic, and endoscopic findings can closely resemble those of achalasia, the treatment and prognosis often differ.^3^

Breast cancer is among the most prevalent cancers affecting women, with nearly 30% of those diagnosed with early-stage breast cancer progressing to metastatic disease.^4^ The primary sites of metastatic spread commonly include the bones (51%), the lungs (17%), the brain (16%), and the liver (6%).^5^ Esophageal metastasis in breast cancer is highly unusual, estimated to be only roughly 1%–6% of all breast cancer cases and typically manifests after a substantial interval between the initial diagnosis and treatment of the primary breast tumor.^1,6^ Previous findings indicate that the timeline for metastatic spread from a primary breast tumor to the esophagus is highly variable, occurring between 8 and 30 years after the initial diagnosis.^7–9^

This is a rare case of esophageal metastasis secondary to a primary breast carcinoma presenting as pseudoachalasia.

## CASE REPORT

A 65-year-old woman with estrogen-positive and progesterone-positive, HER-2/neu-negative, stage IV (T2N2M1) right breast cancer on anastrozole and palbociclib was admitted to the hospital for oral intake intolerance and mild nausea and vomiting. The patient had previously completed multiple rounds of chemotherapy with Ibrance and Arimidex, as well as 5 years of hormone therapy initially with tamoxifen for 2 years followed by anastrozole. Although her dysphagia was progressive, it was a relatively recent development in context of her cancer therapy, endorsing that she had not experienced any of these symptoms during or succeeding her initial treatment. Furthermore, while her current and previous medications are known to cause nausea, vomiting, oral mucosal sores, and pharyngitis, dysphagia is exceedingly rare and typically occurs only in the context of an allergic reaction, which would likely present with additional symptoms. Therefore, although initially included in the differential diagnosis, it was considered an unlikely cause of her progressive dysphagia.

When the patient was admitted to the hospital, she had increasing difficulty with swallowing. She had progressive inability to consume any solids, and even water would often be forcibly regurgitated. She had an esophageal dilatation performed 2 weeks prior without any significant lasting improvement. Esophageal manometry showed instances of weak fragmented swallowing with panesophageal pressurization (<20%) but normal integrated relaxation pressure (IRP) and intrabolus pressure with complete bolus clearance, which was overall nondiagnostic. The manometric findings described are more suggestive of pseudoachalasia rather than true achalasia for several reasons. In classic achalasia, the defining features on manometry include elevated IRP due to the failure of lower esophageal sphincter to relax adequately during swallowing, as well as absent or markedly impaired peristalsis in the esophageal body. These findings are typically accompanied by incomplete bolus clearance due to esophageal outflow obstruction. By contrast, this case demonstrates normal IRP and intrabolus pressure, which suggests that lower esophageal sphincter's relaxation and bolus transit are intact. In addition, the presence of panesophageal pressurization, though limited in frequency, is unusual in achalasia unless associated with Type II achalasia, where pressurization occurs more consistently due to esophageal compression. The preserved bolus clearance further differentiates this condition from achalasia, as achalasia typically results in significant esophageal stasis and impaired bolus transport. Weak, fragmented swallows with intermittent pressurization suggest either a mechanical obstruction or secondary esophageal dysfunction rather than the primary neurodegenerative process underlying achalasia. Given these findings, additional diagnostic evaluation to identify potential structural or malignant causes was pursued.

**Figure 1.**
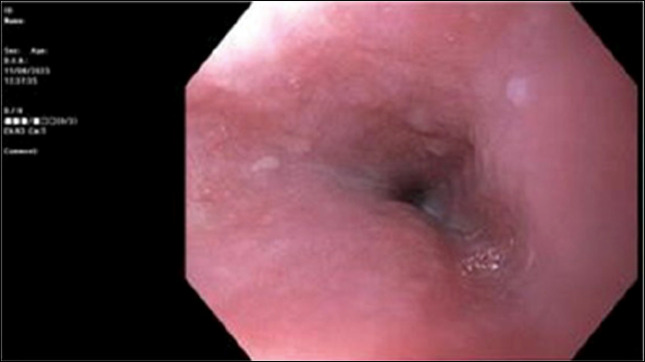
Esophagogastroduodenoscopy with luminal narrowing in distal third of esophagus impeding passage of endoscope despite normal-appearing mucosal surface with no visible intrinsic stricture or lesion.

Upper endoscopy with dilatation was then performed. On initial pass, the endoscope was unable to traverse the distal esophagus despite multiple attempts due to luminal narrowing; however, the mucosal lining appeared normal with no visible intrinsic stricture (Figure 1). When the esophagus relaxed, the endoscope was able to successfully pass allowing for a complete examination and endoscopic balloon dilation up to 15 mm. Biopsies at the gastroesophageal junction showed cardiac-type mucosa with surface hyperplastic changes; however, no dysplasia or cancer was identified by mucosal biopsies.

**Figure 2.**
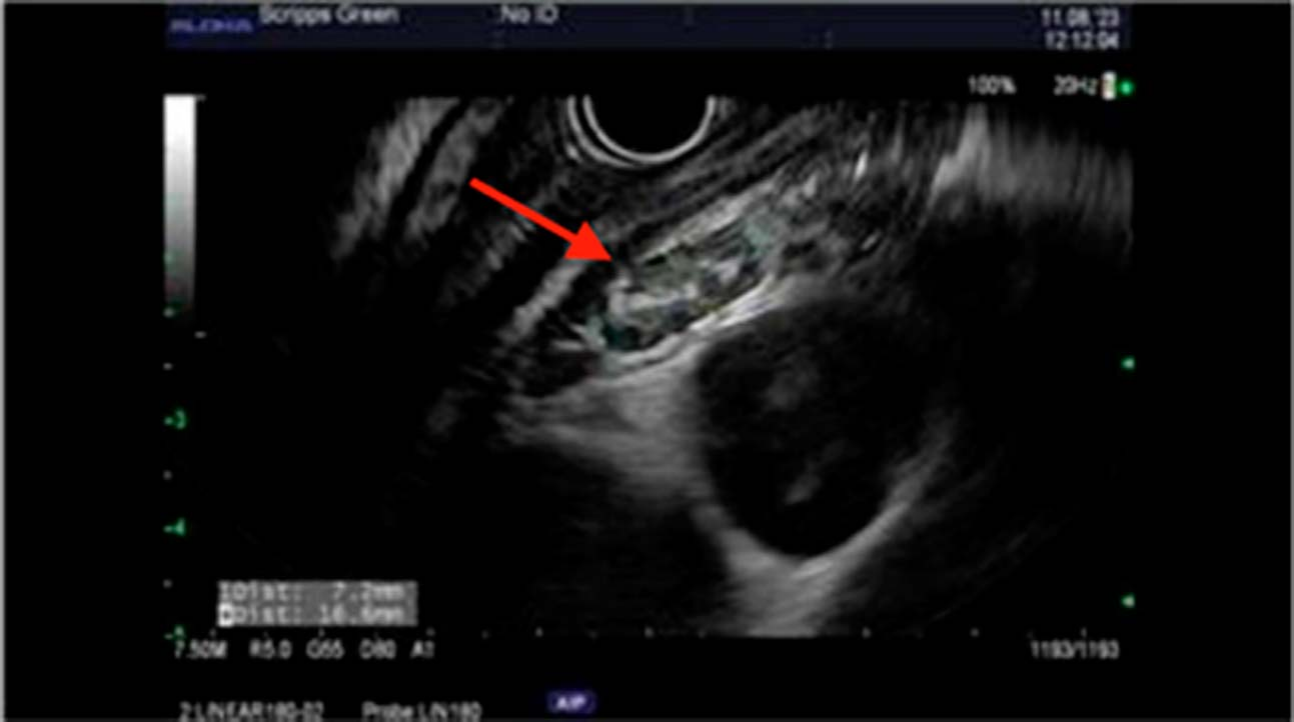
Endoscopic ultrasound notable for circumferential esophageal wall thickening to 8.5 mm. Enlarged lymph node at 25 cm from incisors (red arrow) measured at 18.6 × 6.6 mm status after fine-needle aspiration.

Given concern for possible pseudoachalasia, endosonography (EUS) was then performed. The linear array endoscope was unable to traverse the distal esophagus again due to luminal narrowing. On EUS, the mucosa between 25 and 30 cm from the incisors showed mild circumferential thickening between 6 and 8 mm. The muscular layers appeared preserved in this area. At 26 cm from the incisors, an enlarged lymph node measuring 18.6 × 6.6 mm with a follicular center was detected, along with several smaller lymph nodes in the adjacent area with a similar appearance (Figure 2). EUS fine-needle aspiration (FNA) was performed in the esophagus wall and paraesophageal lymph node using a 25 gauge needle.

The cytology from the esophagus wall and paraesophageal lymph node was suggestive of metastatic carcinoma. Both samples stained positive for AE1/AE3, CK7, GATA3, and GCDFPP15 and mammaglobin compatible with breast cancer. The lymph node aspirate was also found to be estrogen and progesterone positive, as well as HER-2/neu negative consistent with the immunohistochemistry profile of the primary tumor. Her carcinoembryonic antigen was mildly elevated at 6.62 ng/mL.

## DISCUSSION

Timely identification of pseudoachalasia, especially when associated with malignancy, is paramount to prevent mismanagement and ensure timely intervention. However, distinguishing between achalasia and pseudoachalasia can pose significant challenges, as the clinical and diagnostic features often overlap, including that of manometry and radiological imaging, making an accurate diagnosis challenging.^3^ Therefore, it is imperative to be attuned to red flags associated with malignancy including rapid or atypical progression of symptoms, B symptoms such as weight loss, and risk factors such as advanced age.

While computed tomography (CT) imaging holds potential for identifying a neoplastic lesion causing pseudoachalasia, its diagnostic efficacy varies, with malignancy detection reported in only a subset of cases.^3^ However, CT imaging may show irregular or asymmetric wall thickening of distal esophagus, nodularity of the gastroesophageal junction, and possibly a well-defined mass or even metastatic disease, which if seen can be instrumental in narrowing down a differential diagnosis.^10,11^ Endoscopic evaluation remains foundational in the diagnostic approach, especially when excluding obstructions associated with malignancy. It serves as a reliable method for identifying esophageal tumors, strictures, webs, or rings. Careful endoscopic examination including retroflexion for complete examination of the gastric cardia is required in evaluation for pseudoachalasia. Abnormal endoscopic findings, such as nodularity, resistance when passing the GE junction, ulceration, or bleeding, raise suspicion for malignant process, but there may be cases where the endoscopic findings are subtle, especially with pseudoachalasia due to extrinsic compression or an infiltrative process in the esophageal wall. This requires a high index of suspicion to refer patients for chest imaging or EUS to evaluate the esophageal wall and surrounding structures. Endosonography is the best modality for assessment of the esophageal wall layers including thickness of the muscle layer or submucosal infiltration. In this particular case, EUS with FNA was required to make the diagnosis of pseudoachalasia due to metastatic breast cancer to the esophagus.

Treatments for pseudoachalasia due to malignancy are primarily tumor-directed strategies. Surgical excision with or without chemotherapy or radiation therapy represents the mainstay of treatment when possible. In cases where cure is unattainable, palliative measures such as esophagus stenting can effectively alleviate symptoms and enhance the patient's quality of life.

This case adds to the growing body of literature describing malignancy-associated pseudoachalasia, highlighting its diagnostic challenges and the critical role of endosonography. While pseudoachalasia due to malignancy has been reported in association with esophageal, gastric, pancreatic, and lung cancers, breast cancer metastasizing to the esophagus and mimicking achalasia is exceedingly rare. Compared with previously reported cases of pseudoachalasia caused by malignancies, this case is unique in that it is the first reported case of metastatic breast cancer causing pseudoachalasia identified by EUS. While prior reports suggest that CT imaging can sometimes identify malignancy-related esophageal thickening, and endoscopy can provide diagnosis, our case required EUS with FNA for definitive diagnosis, emphasizing the limitations of conventional measures and highlighting the invaluable role of EUS.

In summary, breast cancer metastases to the esophagus are very rare, and it is even more uncommon to present as pseudoachalasia. This case reinforces the importance of a high index of suspicion for malignancy in patients presenting with atypical achalasia features, particularly those with a history of cancer. It also underscores the pivotal role of EUS in evaluating suspected pseudoachalasia when standard imaging, and endoscopy remain inconclusive. Recognition of malignancy-associated pseudoachalasia is crucial. In patients with a history of cancer, risk factors, or atypical presentation of achalasia, there should be a low threshold to consider EUS to rule out pseudoachalasia when standard workup with imaging, endoscopy, and motility testing is nondiagnostic.

## DISCLOSURES

Author contributions: G. Teskey: lead author and is the article guarantor; A. Prevallet: editor; R. Khanna: editor; F. Chung-Han Tsai: investigator/editor; WJ Coyle: lead investigator.

Financial disclosure: None to report.

Informed consent was obtained for this case report.
